# Converging Fate of the Oxidation and Reduction of 8-Thioguanosine

**DOI:** 10.3390/molecules24173143

**Published:** 2019-08-29

**Authors:** Katarzyna Taras-Goslinska, Fabrizio Vetica, Sebastián Barata-Vallejo, Virginia Triantakostanti, Bronisław Marciniak, Chryssostomos Chatgilialoglu

**Affiliations:** 1 Adam Mickiewicz University, Faculty of Chemistry, Wieniawskiego 1, 61-712 Poznań, Poland; 2 R&D Laboratory, Lipinutragen srl, Via Piero Gobetti 101, 40129 Bologna, Italy; 3 ISOF, Consiglio Nazionale delle Ricerche, Via Piero Gobetti 101, 40129 Bologna, Italy; 4 Universidad de Buenos Aires, Facultad de Farmacia y Bioquimíca, Departamento de Quimíca Organíca, Junin 954, RA-1113 Buenos Aires, Argentina; 5 Center of Advanced Technologies, Adam Mickiewicz University, 61-712 Poznań, Poland

**Keywords:** photolysis, laser flash photolysis, γ-radiolysis, singlet oxygen, nucleosides, free radicals, reaction mechanism

## Abstract

Thione-containing nucleobases have attracted the attention of the scientific community for their application in oncology, virology, and transplantology. The detailed understanding of the reactivity of the purine derivative 8-thioguanosine (8-TG) with reactive oxygen species (ROS) and free radicals is crucial for its biological relevance. An extensive investigation on the fate of 8-TG under both reductive and oxidative conditions is here reported, and it was tested by employing steady-state photooxidation, laser flash photolysis, as well as γ-radiolysis in aqueous solutions. The characterization of the 8-TG T_1_ excited state by laser flash photolysis and the photooxidation experiments confirmed that singlet oxygen is a crucial intermediate in the formation of the unexpected reduced product guanosine, without the formation of the usual oxygenated sulfinic or sulfonic acids. Furthermore, a thorough screening of different radiolytic conditions upon γ-radiation afforded the reduced product. These results were rationalized by performing control experiments in the predominant presence of each reactive species formed by radiolysis of water, and the mechanistic pathway scenario was postulated on these bases.

## 1. Introduction

The search for new purine and pyrimidine derivatives of biological significance has been conducted by many research groups [1,2]. The analogues of purine bases of nucleic acids containing a sulfur atom, such as mercaptopurine, 6-thioguanine, or azathioprine, have been applied in medicine for many years in the treatments of cancer, in particular leukemia in children, viral diseases, and in transplantology [3,4,5]. However, it has been recently evidenced that the skin of patients taking these drugs for a long time becomes much more sensitive to sunlight, and the chances of skin cancer development in these patients increase [6,7]. A small structural modification involving the attachment of a thione group to the purine ring system results in a decrease in energy of the electronic excited states, leading to absorption of long-wavelength light, including the UV-A range [8]. Moreover, thiocarbonyl compounds in excited states are highly reactive [9]. Therefore, thio-derivatives of guanine present in the chains of nucleic acids can take part in photochemical transformations or at least can initiate them. The photochemical processes upon excitation of thioguanosine chromophore in DNA include oxidation of DNA bases and/or proteins by ^1^O_2_ or other reactive oxygen species (ROS), photo cross-linking of DNA strands, and generation of reactive radicals that can modify the components of nucleic acids or proteins. In the photochemical studies of 6-thioguanosine (6-TG), most attention has been paid to the oxidation reactions, as they are assumed to be mainly responsible for the adverse biological effects [10,11]. It has been reported that in aerated aqueous solutions, after absorption of UV-A, 6-TG undergoes Type I and Type II photooxidation processes and generates ROS, including singlet oxygen ^1^O_2_ or radicals, harmful to biological systems [12,13]. After selective excitation, 6-TG built in DNA generates singlet oxygen ^1^O_2_ through energy transfer from the excited electronic state of the 6-TG molecule to the ground state oxygen molecule. Other reactive individuals formed in the presence of oxygen include radicals generated in the process of electron transfer or charge–transfer complexes between the triplet state of 6-TG and ^3^O_2_ [8,12].

Guanine substituted at the C8 position with either a bromine or thiol group is a potent adjuvant [14]. As an approach to reveal the photooxidation mechanism, we have started the investigations on the properties of 8-thioguanosine (8-TG, **1**) excited states by steady-state and time-resolved techniques. The electronically excited 8-TG molecule in the presence of oxygen generates reactive species, including singlet oxygen ^1^O_2_ or radicals. 

**Scheme 1:**
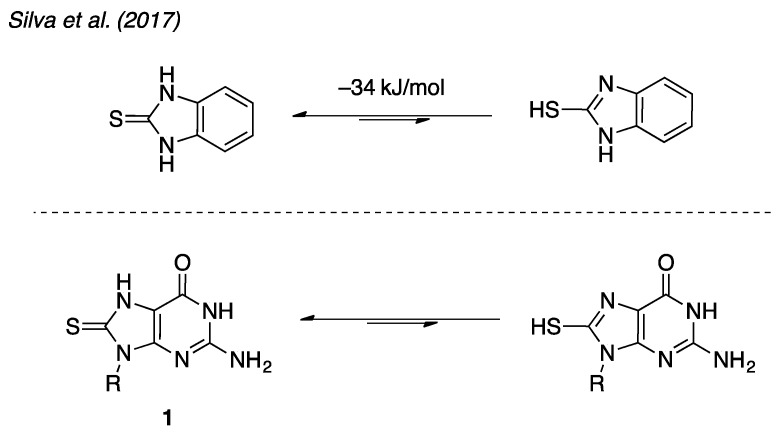
Tautomeric equilibrium of 8-thioguanosine (8-TG).

On a structural point of view, the presence of a sulfur atom at the C8 position in 8-TG results in the establishment of a tautomeric equilibrium between the thione and the thiol form. Equilibrium of mercapto-azoles has attracted interest in both experimental and theoretical chemistry because of the relevance of this molecule in biological applications. Theoretical studies on 2-mercaptobenzimidazole, with a similar heterocyclic scaffold to 8-TG, revealed that the equilibrium favors the thione form (tautomerization energy thiol/thione is −34.2 kJ/mol) [15]. Thus, it is reasonable to consider that 8-TG would favor the thione form as well (Scheme 1).

Therefore, 8-thioguanosine could potentially have a similar reactivity as the thioureas, specifically cyclic thioureas. The thiourea scaffold has been widely investigated over the years for its applications in synthetic organic chemistry, industry [16], medicinal chemistry, and agrochemistry [17], and as an ROS scavenger [18,19]. Because of the highly applicability, several research groups have been interested in the redox chemistry of thioureas and have reported detailed mechanistic studies for these transformations. The reactivity of thioureas with hydrogen peroxide [20], common reducing agents such as K and NaBH_4_ [21], singlet oxygen [22], and upon pulse radiolysis in both reducing and oxidizing conditions have been studied [23,24,25,26,27,28,29], outlining multiple mechanistic scenarios. 

With these premises, in this study we report our investigation on the steady-state photooxidation and laser flash photolysis of 8-TG, as well as its γ-radiolysis in aqueous solution, under both reductive and oxidative conditions, highlighting an unusual and converging product formation.

## 2. Results and Discussion

### 2.1. Structural Characterization of 8-Thioguanosine (8-TG)

Initially, we decided to register NMR spectra in order to define which tautomeric form of 8-TG is favored. The proton NMR in DMSO-d_6_ shows 2 different broad signals at 11.13 and 12.63 ppm, corresponding to NH from amide and thioamide moieties (Figures S1–S3). Moreover, both peaks exchange after adding D_2_O. These results, combined with the previously reported theoretical studies (vide supra), suggest that the equilibrium favors the thione form.

**Figure 1:**
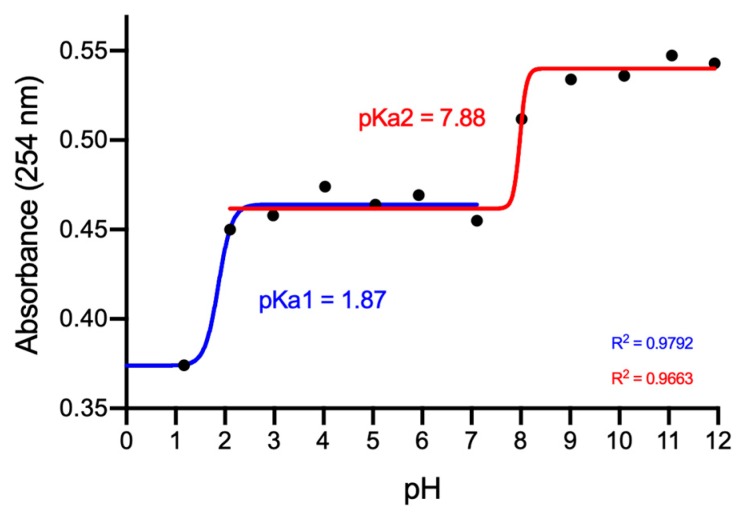
UV-pH titration of 8-TG.

Additionally, we decided to perform a UV titration of aqueous solutions of 8-TG to calculate the p*K*_a_ value. Using 12 different 0.1 mM solutions of 8-TG, each at a different pH (using NaOH/phosphoric acid buffers), we recorded the absorption values and we plotted the absorption versus pH of the solution (Figure 1). The resulting data were fit into sigmoid curves, and the inflection points were calculated. The titration curve showed 2 p*K*_a_ values: p*K*_a1_ = 1.87 and p*K*_a2_ = 7.88, which presumably correspond to NH_2_ protonation and deprotonation of the amide moiety, respectively [30,31].

### 2.2. Steady-State Photochemistry 

**Figure 2:**
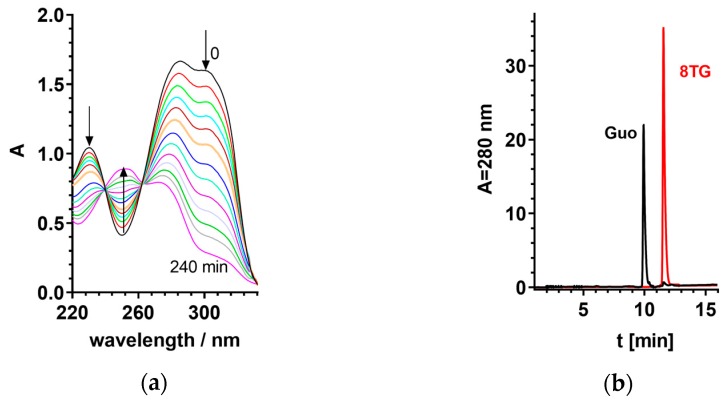
(**a**) UV spectra of 8-TG (c = 1.8 × 10^−4^ M) in air-equilibrated aqueous solution before (black) and after 240 min. Irradiation at λ = 313 nm, (**b**) Chromatogram of the solution of 8-TG before (red) and after irradiation (black).

**Scheme 2:**
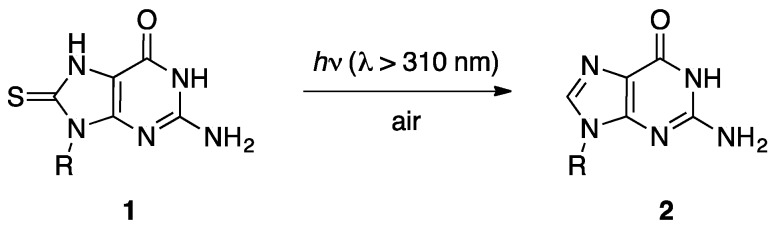
The formation of Guo (**2**) upon excitation of 8-TG (**1**).

Direct excitation of 8-TG in air-equilibrated aqueous solution with λ > 300 nm light or monochromatic radiation at 313 nm leads to disappearance of the intense absorption band (λ_max_ = 284 nm, ε_max_ = 18,700 M^−1^·cm^−1^) of the substrate and increase in absorption in the range of 240–260 nm (Figure 2a). The spectral changes are compatible with the expectation that the thiocarbonyl group is engaged in the photoreaction. The HPLC analysis revealed 88.5% conversion of the substrate and a formation of guanosine (Guo, **2**) as the major photoproduct (Figure 2b). The product was formed with a chemical yield of 89% based on the substrate reacted (Scheme 2).

**Figure 3:**
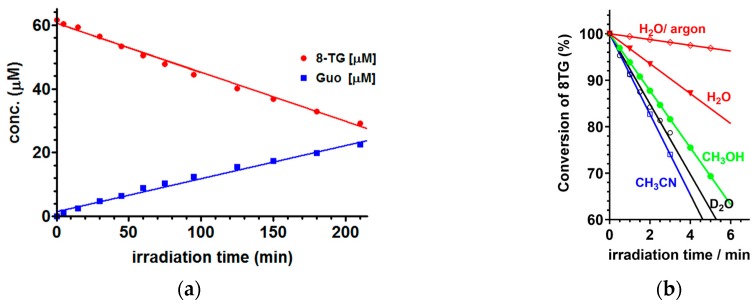
(**a**) The concentration of 8-TG (red) and Guo (blue) as a function of the irradiation time in air-equilibrated aqueous solution. (**b**) Rates of substrate disappearance upon radiation at λ > 300 nm of 8-TG in air-equilibrated solutions with different solvents.

Guo (**2**) was found to be the major photoproduct under very low (<15%) conversion of 8-TG as well (Scheme 2). The UV absorption spectra recorded during the irradiation and chromatogram of the irradiated solution are presented in Figure 2. The product concentration profile showed that it was present at very low substrate conversion (Figure 3a). Therefore, it did not arise from secondary photochemistry.

In contrast to the efficient photochemistry in the presence of atmospheric oxygen, the disappearance of the substrate was slowed down by a factor of 10 when aqueous solution was purged by argon for 15 min prior to irradiation (Figure 3b). Several products were observed, but guanosine was not identified. Thus, the presence of oxygen is required for the photochemical transformation of 8-TG to Guo, suggesting that the observed transformation is a photooxidation reaction. The nature of the solvent has an impact on the rate of 8-TG disappearance (Figure 3b). A higher rate was observed in aerated organic solvents. Moreover, the photochemical reaction rate of 8-TG in D_2_O increased twofold as compared to the rate determined in aqueous (H_2_O) solution.

In general, two mechanisms of photochemical oxidation of organic compounds can be distinguished: a mechanism (type II) with the involvement of singlet oxygen (^1^O_2_), a highly reactive oxygen molecule in its excited electronic state, and a nonsinglet oxygen mechanism (type I) [7,32].

**Figure 4:**
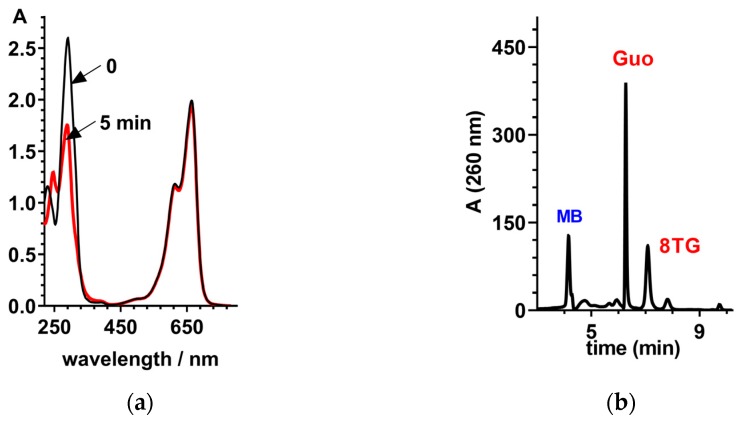
(**a**) UV absorption spectra recorded in the course of radiation of methylene blue (MB) and 8-TG with λ > 400 nm; (**b**) Chromatogram of the irradiated mixture.

In order to establish which mechanism was operating in the case of 8-TG, the compound was subjected to a reaction with ^1^O_2_. Methylene blue (MB) and rose bengal (RB) are very effective sensitizers of ^1^O_2_ upon visible light irradiation in aerobic conditions [33]. Figure 4 shows the absorption spectra of a mixture of MB and 8-TG before and after irradiation (λ > 400 nm) and a chromatogram of the irradiated mixture. Guo was found to be a major product in both MB- and RB-sensitized reactions of 8-TG, with chemical yields of 57% and 54%, respectively.

**Scheme 3:**
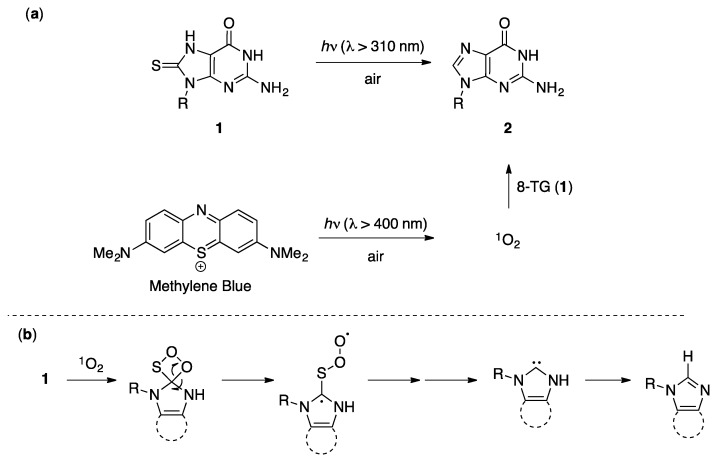
(**a**) Photochemical oxidation of 8-TG and photosensitized generation of singlet oxygen; (**b**) Proposed mechanistic pathway of the ^1^O_2_-promoted desulfurization of 8-TG.

The formation of Guo upon excitation of 8-TG with λ = 313 nm light and in a reaction sensitized by dyes (λ > 400 nm) suggests that the reactive oxygen species, ^1^O_2_, is involved in the direct photooxidation of 8-TG (Scheme 3a). The generation of ^1^O_2_ by 8-TG, like in the case of 6-TG, may occur via an energy transfer from the 8-TG triplet excited state (T_1_) to the oxygen molecule in the electronic ground state. 

The desulfurized substrate is an unexpected photoproduct obtained from photooxidation of heteroaromatic thiocarbonyl compounds [34,35]. The photoproduct molecules identified so far always contained oxygen atom(s). The major photoproducts were: ketones, sulfines, and, in the case of enolizable thiones, sulfinates and sulfonates. For example, the photoproducts obtained from 6-thioguanine, under experimental conditions identical to that applied for 8-TG, have been identified as guanine 6-sulfinate, guanine 6-sulfonate, and guanine as a minor photoproduct [36]. Comprehensive theoretical and experimental investigations of 6-TG photoxidation performed by Zou et al. have shown that all stable isolated photoproducts, most likely, derive from a common, unstable peroxy intermediate 6-(SOOH)-G [37]. 6-(SOOH)-G is suggested to be a primary product of the ^1^O_2_ attack on sulfur atoms in the 6-TG molecule. 

Oxygenated sulfur compounds, like sulfinic and sulfonic acids, as well as the carbonyl analog of 8-TG, have not been detected. Considering what has been previously described for 6-TG and the proposed mechanism hypothesized for thiourea in the presence of singlet oxygen [22], we propose that a similar [2 + 2] attack of ^1^O_2_ on the thione moiety would lead to a thioperoxyl radical via 4-membered-ring opening. Subsequently, this unstable intermediate could, instead of proceeding to an oxygenated sulfur compound, evolve to form a carbene, leading afterwards to guanosine (**2**) (Scheme 3b). 

### 2.3. Laser Flash Photolysis 

It has been established that the excited triplet state plays a key role in the generation of singlet oxygen. That is why the characterization of this state, configuration, radiative and radiation-less pathways of deactivation, and yield of singlet oxygen generation is so important [38]. 

**Figure 5:**
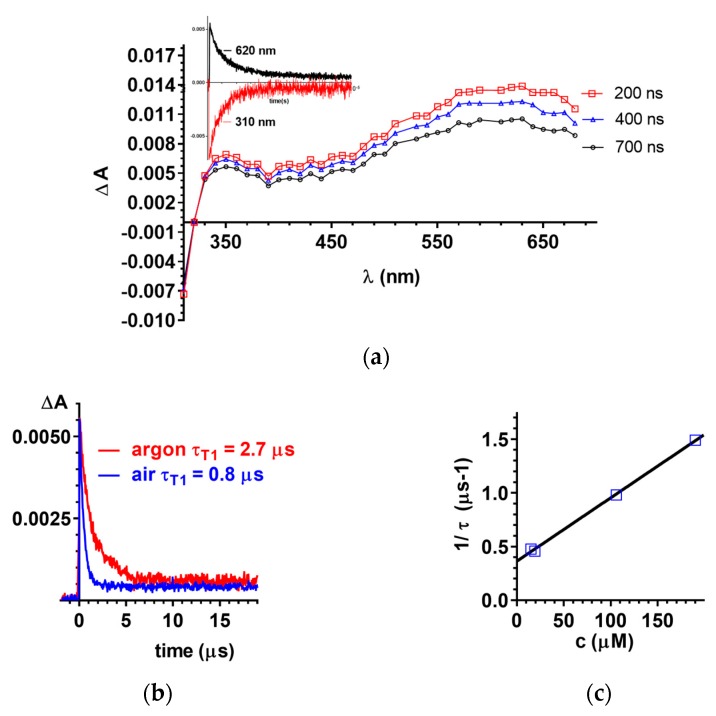
(**a**) Transient absorption spectrum of 8-TG (0.62 mM) in argon-saturated aqueous solution at various delay times (λ_exc_ = 266 nm, 3 mJ). Insert: kinetic traces of the 620 nm absorbance decay and 310 nm the ground state depopulation of 8-TG in argon-saturated aqueous solution; (**b**) Kinetic traces of the 620 nm absorbance decay in argon-saturated and air-equilibrated aqueous solution; (**c**) Stern–Volmer plot of reciprocal of triplet lifetime vs. concentration of 8-TG in argon-saturated solution.

The properties of the lowest energy excited states of 8-TG have been studied by laser flash photolysis. In argon-saturated aqueous solution, only transient absorption of the excited triplet state (T_1_) of 8-TG can be observed in nanosecond and microsecond time scales. Transient absorption spectra of 8-TG in Ar-saturated aqueous solution are shown in Figure 5a. Immediately after the laser, a broad spectrum extending over 330–750 nm was observed. From these kinetic traces, it was found that the decay of T_1_ was independent of the monitoring wavelength, suggesting that the two bands can be assigned to a single excited species. Single exponential fits were made for the transient absorption decays measured for a series of 8-TG solutions having concentrations in the range of 0.05–0.5 mM. The determined rate constants were then used in the Stern–Volmer plot. Under these conditions, the T_1_ state of 8-TG was relatively long-lived. The intrinsic (concentration-independent) T_1_ state lifetime, τ^0^ = 3.6 μs, was determined from the Stern–Volmer plot (Figure 5c). The T_1_ state is quenched rapidly by the ground state O_2_ molecules with a rate constant k_Q_ = 5 × 10^9^ M^−1^·s^−1^ (in air-equilibrated aqueous solution). The kinetic traces of the T_1_ state of 8-TG in argon-saturated and air-equilibrated aqueous solution, respectively, determined by monitoring the absorption decay at λ = 620 nm are presented in Figure 5b. The primary photochemical processes in aqueous solution are triplet state formations, as shown by flash photolysis.

Since 8-TG (T_1_)* is quenched by dissolved molecular oxygen, a sensitization reaction between 8-TG* and molecular oxygen should occur [39]. In the presence of oxygen, radicals can also be formed in the process of electron transfer. In particular, evidence for presence of the 8-TG radical or radical cation was sought in transient absorption measurements. The transient absorption spectrum of 8-TG obtained under aerated conditions is identical to the spectrum recorded in the Ar-saturated solution.

**Figure 6:**
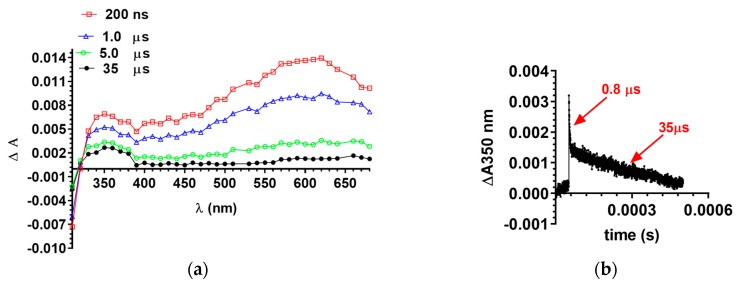
(**a**) Transient absorption spectrum of 8-TG (0.62 mM) in air-equilibrated aqueous solution at various delay times (λ_exc_ = 266 nm, 3 mJ). (**b**) Kinetic trace of the 340 nm absorbance decay in air-equilibrated aqueous solution.

However, the compared decay dynamics of the T_1_ state at 340 nm shows the appearance of a new transient. We observed a long-lived transient (λ_max_ = 340 nm) formed in reaction of 8-TG with oxygen. Based on our experience and literature reports we can assume that the observed species is a radical or radical cation. (Figure 6). The transient was found to disappear 35.0 µs after the laser pulse, it was not quenched by oxygen, and the recorded spectrum (λ_max_ = 350 nm) was in the range where guanosine radicals were previously observed [31]. 

For 8-TG, the radical processes have not been observed so far, but they are well known for guanine [40]. Molecular oxygen is known to act as an electron acceptor whether it is in its ground state or in its excited singlet state [41]. Both processes are presented below with the participation of 8-TG in its T_1_ excited state or in its ground state (S_0_).

8-TG(T_1_) + ^3^O_2_ → 8-TG^+•^ + O_2_^•^, (1)

8-TG(S_0_) + ^1^O_2_ → 8-TG^+•^ + O_2_^•−^, (2)

### 2.4. γ-Radiolysis of 8-TG in Aqueous Solutions

Radiolysis of neutral water leads to the reactive species e_aq_^−^, HO^•^, and H^•^, together with H^+^ and H_2_O_2_, as shown in Equation (3). The values in brackets represent the radiation chemical yield (*G*) in µmol·J^−1^ [42].

H_2_O + γ-irr. → e_aq_^−^(0.27), HO^•^(0.28), H^•^(0.06), H^+^(0.06), H_2_O_2_(0.07) (3)

Having discovered the unexpected reductive desulfurization of 8-TG in oxidative conditions, our investigation proceeded with the γ-radiolysis of **1** in the presence, initially, of all the reactive species generated by radiolytic decomposition of water: e_aq_^−^, HO^•^, and H^•^. Irradiations were performed at room temperature using a ^60^Co-Gammacell at different doses. The exact absorbed radiation dose was determined by employing the Fricke chemical dosimeter, by taking *G*(Fe^3+^) = 1.61 µmol·J^−1^ [43].

**Figure 7:**
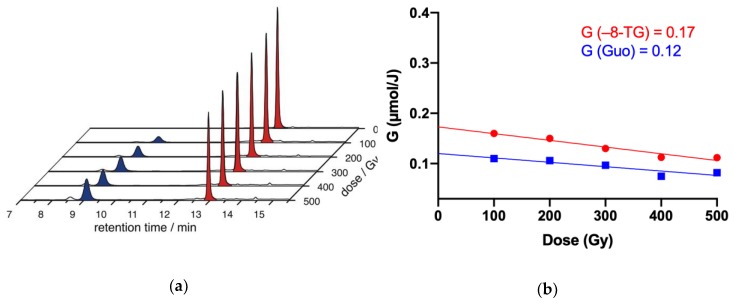
γ-radiolysis of **1** (0.196 mM) in N_2_-purged water at a natural pH at a dose rate of 1.85 Gy·min^−1^. (**a**) HPLC runs of the reactions. The HPLC peaks of **1** are highlighted in red, while the peaks of guanosine (**2**) are highlighted in blue. (**b**) The chemical irradiation yields *G* (−**1**) (**●**) and *G* (**2**) (**■**) as a function of the irradiation dose. The line extrapolated to the zero dose leads to the *G* values reported in the graph.

We investigated this reaction by detailed product studies in a dose-dependent experiment, irradiating 6 different independent samples (each 0.196 mM of **1**) for 100 Gy dose intervals, until achieving a total dose of 500 Gy. The reaction mixtures were monitored by HPLC-UV (254 nm) analysis, and the chromatograms were plotted in Figure 7a. In the dose course, we could observe consistent consumption of the starting material **1** (red peak, RT = 13.01 min) and simultaneous formation of the main product (blue peak, retention time (RT) = 9.8 min). Mass spectra of this product showed the mass of guanosine **2**, also confirmed by comparison with commercially available guanosine as well as spiking experiments. The product yield (mol·kg^−1^) divided by the absorbed dose (1 Gy = 1 J·kg^−1^) gives the reaction’s chemical yield (*G*). The *G* values at each analyzed dose were calculated and plotted with the dependence of the dose (Figure 7b). The radiation chemical yields extrapolated to the zero dose are: *G* (−**1**) = 0.17 and *G* (**2**) = 0.12 µmol·J^−1^. Considering the sum of the *G* values of the reactive species (Equaction (3)) of 0.61 µmol·J^−1^ (0.68 if we consider H_2_O_2_ contribution), we could observe that 0.44 (or 0.51 respectively) of the reacting radicals returned to starting materials, while 0.17 reacted productively with **1**. Moreover, 70.6% of these radicals (*G* = 0.12 µmol·J^−1^), lead to the formation of product **2**. Unknown products formed and were visible in the formation of small peaks at RT = 8.6 and 14.45 min.

The results obtained in the above γ-radiolysis experiment and in the absence of oxidation products, such as 8-oxo-Guo or the disulfide dimer of 8-TG, underline an unexpected and more complex mechanistic scenario that is in accordance with the results obtained in the photooxidation experiments. Therefore, we decided to investigate some aspects of these reactions in more detail. 

Before moving forward to the reactive species, we decided to first test the reactivity of **1** with H_2_O_2_, which is also formed by radiolysis of water. Indeed, the reaction of thiourea derivatives with hydrogen peroxide is a well-known desulfurization process, leading to the reduced product and SO_2_ [20]. Therefore, we carried out an experiment outside the Gammacell using an aqueous solution of 8-TG (0.3 mM) in the presence of 10% H_2_O_2,_ kept without stirring for 4.5 h, and a time comparable to 500 Gy if the reaction was done in Gammacell (Figure S12, Supporting Information). As expected, the formation of Guo (**2**) as a major product was observed.

#### 2.4.1. γ-radiolysis of 8-TG in Aqueous Solutions under Reductive Conditions

Afterwards, we focused first on the reactivity of e_aq_^−^ and H^•^ with **1**, employing N_2_-saturated water solutions at a natural pH. *t*BuOH (0.25 M) was added to the reaction mixture in order to suppress the HO^•^ generated from the radiolytic decomposition of water (Equation (4)). In fact, efficient conversion of hydroxyl radicals into the less reactive *tert-*butyl radicals allows studying the reaction of the e_aq_^−^ and H^•^ atoms because the presence of *t*BuOH also suppresses H^•^ radicals, but only slightly since the reaction rate is more than three orders of magnitude lower (Equation (5)) [42].

HO^•^ + *t*BuOH → (CH_3_)_2_(OH)CH_2_^•^ + H_2_O   (*k* = 6.0 × 10^8^ M^−1^·s^−1^) (4)

H^•^ + *t*BuOH → (CH_3_)_2_(OH)CH_2_^•^ + H_2_   (*k* = 1.7 × 10^5^ M^−1^·s^−1^) (5)

**Figure 8:**
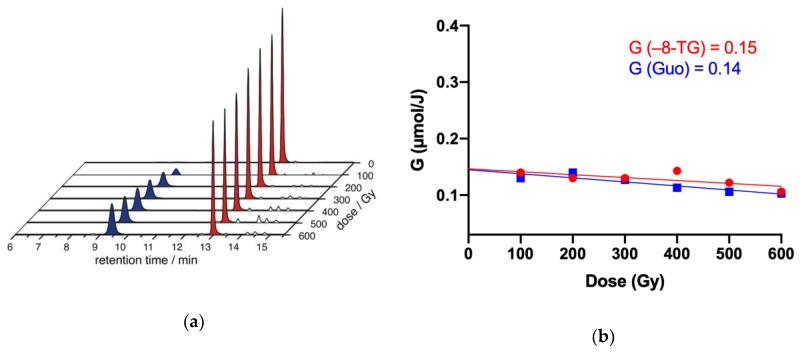
γ-radiolysis of **1** (0.25 mM) in N_2_-purged water at a natural pH, containing *t*BuOH (0.25 M) at a dose rate of 1.85 Gy·min^−1^. (**a**) HPLC runs of the reactions. The HPLC peaks of **1** are highlighted in red, while the peaks of guanosine (**2**) are highlighted in blue. (**b**) The chemical irradiation yields *G* (−**1**) (**●**) and *G* (**2**) (**■**) as function of the irradiation dose. The line extrapolated to the zero dose leads to the *G* values reported in the graph.

From Figure 8a we can infer that the gradual disappearance of 8-TG occurred with the formation of Guo (**2**). Furthermore, no evidence of any side reaction was found, a part from some negligible peaks eluted after the starting material (RT between 14 and 15 min). The *G* values at each analyzed dose were calculated and plotted with the dependence of the dose (Figure 8b). The radiation chemical yields extrapolated to the zero dose are: *G* (−**1**) = 0.15 and *G* (**2**) = 0.14 µmol·J^−1^. These values underline that all the reacted 8-TG was converted solely to product **2** or returned to the starting material and, in a similar scenario, to the previous experiments without *t*BuOH. 

**Scheme 4:**
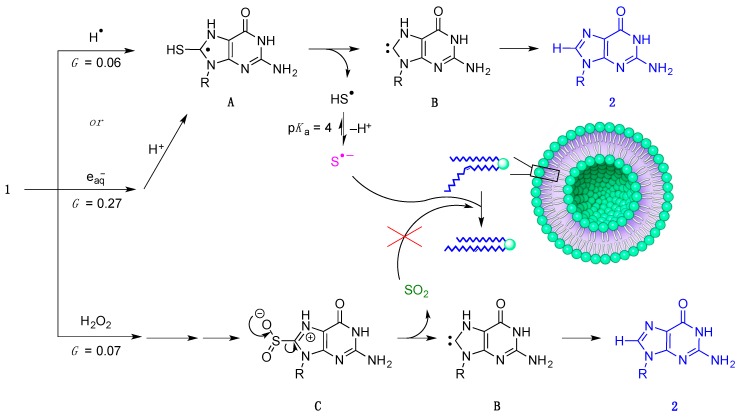
Postulated mechanism of desulfurization promoted by H^•^ atoms and H_2_O_2_.

On the basis of these and the previous results, our mechanistic hypothesis is depicted in Scheme 4. We postulated that the contribution of H_2_O_2_ would proceed, as reported in literature [20], via formation of the oxygenated intermediate **C**, which eliminated SO_2_ and generated a carbene, which then led to product **2**. On the other hand, H^•^ radicals could add on the thione moiety of 8-TG, forming the radical **A**, as suggested by previously reported studies [25]. Subsequently, this species could eliminate HS^•^ radicals, forming the same carbene **B** formed with H_2_O_2_ and achieving Guo (**2**). The combined *G* values of H^•^ radicals and H_2_O_2_ correspond to 0.13 µmol·J^−1^ and the *G* (**2**) = 0.12 µmol·J^−1^; hence, they were in accordance with the suggested pathway. However, the reaction of e_aq_^−^/H^+^ should also potentially contribute to the formation of intermediate **A**.

Concerning the experiment suppressing HO^•^ radicals, on the basis of the observed similar *G* values, we postulated that the reaction could involve H^•^ atoms and H_2_O_2_ (Scheme 4) as well. Indeed, both these species are present in the reaction medium. However, we were interested to also understand the involvement of e_aq_^−^ in the formation of Guo (**2**). The reaction of 8-TG with e_aq_^−^ could generate a radical anion, which, upon protonation, could afford the previously discussed radical formed by the attack of H^•^ atoms. Therefore, the contribution of both H^•^ radicals and/or e_aq_^−^/H^+^ would provide the formation of Guo (**2**) via elimination of HS^•^ and formation of carbene **B**.

With these premises, to confirm whether the mechanistic pathway of this desulfurization proceeded via heterolytic bond cleavage or by thiyl radical formation, we performed the same three experiments in the presence of 1-palmitoyl-2-oleoyl-sn-glycero-3-phosphocholine (POPC) liposomes (2 mM) on the basis of our extensive experience in lipid *cis*/*trans* isomerization catalyzed by thiyl radicals (Scheme 4) [44,45,46]. In fact, if the mechanism involves the elimination of sulfhydryl radicals, we should observe isomerization, while no isomerization should occur if SO_2_ is produced. After 4.5 h reaction time or 500 Gy irradiation, the phospholipids were extracted, transesterified to the methyl esters in methanolic KOH (0.5 M) solution, and analyzed via gas chromatography (GC) (for details see 3.6–3.8). GC analysis of the fatty acid methyl esters revealed that no isomerization occurred in the reaction with H_2_O_2_, supporting our hypothesis, which involves the formation of SO_2_ (Figure S12). Conversely, in the γ-radiolysis experiment we observed 4.5% of isomerization, supporting the postulated elimination of HS^•^ radicals (Figure S7). Moreover, the reaction performed under reductive conditions achieved the desulfurized product **2** while providing isomerization of the oleic double bonds up to 13.7% (Figure S8). 

#### 2.4.2. The Role of HO^•^ Radicals and Other Oxidants

In a subsequent experiment, we deemed it proper to study the reaction of **1** with HO^•^ radicals. In doing so, we performed radiolysis of **1** in N_2_O-saturated water on the basis of the previously discussed transformation. In N_2_O-saturated solution at a natural pH (≈0.02 M of N_2_O), e_aq_^−^ are efficiently transformed into HO^•^ radicals via Equation (6) (*k* = 9.1 × 10^9^ M^−1·^s^−1^), affording *G* (HO^•^) = 0.55 µmol·J^−1^; that is, HO^•^ and H^•^ account for 90% and 10%, respectively, of the reactive species [42].

e_aq_^−^ + N_2_O + H_2_O → HO^•^ + N_2_ + HO^−^   (*k* = 9.1 × 10^9^ M^−1^·s^−1^) (6)

**Figure 9:**
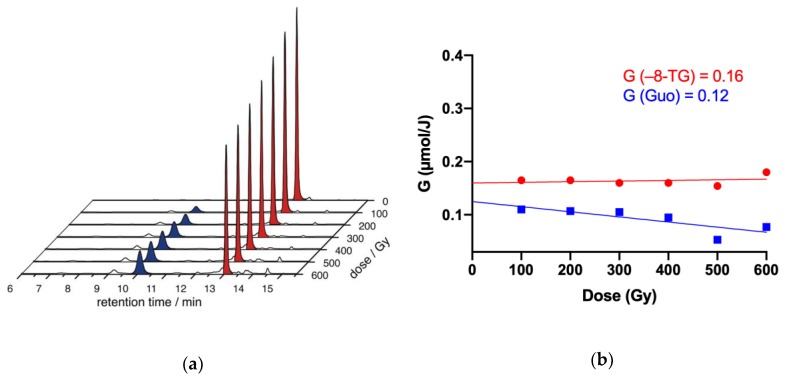
γ-radiolysis of **1** (0.3 mM) in N_2_O-purged water at a natural pH at a dose rate of 1.85 Gy·min^−1^. (**a**) HPLC runs of the reactions. The HPLC peaks of **1** are highlighted in red, while the peaks of guanosine (**2**) are highlighted in blue. (**b**) The chemical irradiation yields *G* (−**1**) (**●**) and *G* (**2**) (**■**) as function of the irradiation dose. The line extrapolated to a zero dose leads to the *G* values reported in the graph.

Solutions of **1** (0.3 mM) were irradiated and stopped at intervals of 100 Gy, up to 500 Gy, and the results are plotted in Figure 9a. Also in this experiment, we observed a gradual consumption of **1** (red peaks) with concurrent formation of the reduction product (**2**, blue) despite the oxidative conditions. At a dose of 600 Gy the product yield was 14%, while the conversion was 54%. However, in this case, we observed the formation of 2 additional peaks, one with RT of ≈9 min and the other ≈14.5 min, which could explain the lower conversion measured. The radiation chemical yields extrapolated at zero dose from the plots in Figure 9b were: *G* (−**1**) = 0.16 and *G* (**2**) = 0.12 µmol·J^−1^. Assuming that these results derive as before from the contribution of H^•^ radicals and H_2_O_2_, since *G* (HO^•^) is 0.55 µmol·J^−1^, the arising question is what happened to HO^•^ radicals since we did not observe further consumption of the substrate **1**.

To answer this question, we decided to employ different oxidant species generated from the conversion of hydroxyl radicals (from Equation (6)), depending of the type of additive present in an N_2_O-saturated solution. In particular, we decided to test the reactivity of **1** in the presence Br_2_^•−^ and N_3_^•^, formed via Equations (7) and (8) from KBr (0.1 M) and NaN_3_ (0.1 M) (respectively *G* (Br_2_^•−^) and *G* (N_3_^•^) = 0.55 µmol·J^−1^).

HO^•^ + N_3_^−^ → N_3_^•^ + HO^−^   (*k* = 1.2 × 10^10^ M^−1^·s^−1^) (7)

HO^•^ + 2Br^−^ → Br_2_^•−^ + HO^−^   (*k* = 1.1 × 10^10^ M^−1^·s^−1^) (8)

In Figure S5 the HPLC-UV runs for the dose-dependent experiments in the presence of N_3_^•^ are depicted. The main product formed was the reduced product guanosine (**2**) at a 33.6% yield and 95.7% conversion after 600 Gy irradiation. From the dose course studies, we could observe the gradual consumption of **1** while **2** consistently formed. The radiation chemical yields highlight that about half of N_3_^•^ radicals reacted with **1** in a productive way, while 66% of these contributed to formation of the product. Interestingly, the same behavior was observed by using Br_2_^•−^, which measured similar *G* values and an identical difference between *G* (−**1**) and *G* (**2**) of 0.07 µmol·J^−1^ (Figure S4).

**Scheme 5:**
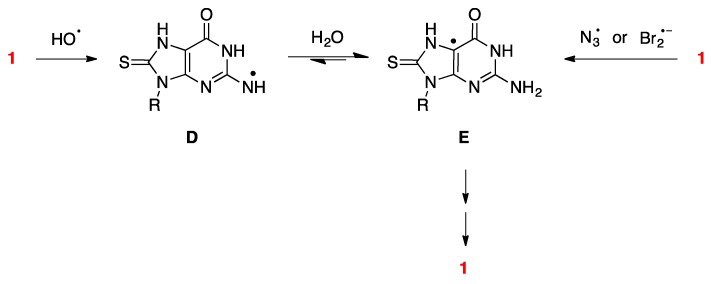
Hypothesized mechanism of the reaction between HO^•^, Br_2_^•−^ and N_3_^•^, forming radical intermediates and leading back to the substrate.

With these results in hand, we were interested in understanding the involvement of these oxidant species in the overall desulfurization of 8-TG (**1**). In fact, in all the reactions we could observe similar results to the ones obtained in the previous experiments in terms of *G* values, while no further consumption of the substrate was achieved via side product formation. On the basis of our strong background of radical chemistry of guanosine derivatives [31,47,48,49,50,51,52], it was established that HO^•^ radicals could perform hydrogen abstraction predominantly on the exocyclic NH_2_ group [48,49,50], leading to the radical intermediate **D** (Scheme 5). This species could undergo tautomerization to the more stable radical **E**. We proved that this tautomeric equilibrium was not affected by substitution at the 8-position of the guanine moiety with H, Br, or alkyl group, and it occurred at a constant of about 3 × 10^4^ s^−1^ [50]. Hence, we could assume that this could be the case as well for 8-TG-derived radical **D**. Once the tautomeric radical **E** formed, this could lead back to the starting material **1**. In fact, this has been reported for similar intermediates for the other studied guanosine derivatives, which resulted in regeneration of the substrate with a still unclear mechanism [31,52]. This pathway could explain the absence of further consumption of 8-TG in the predominant presence of HO^•^ radicals. Additionally, the intermediate **E** could be directly formed by Br_2_^•−^ and N_3_^•^ and provide regeneration of **1** in the same fashion, explaining the comparable results in the presence of these two radical species [31,51,52].

Subsequently, we carried out all these experiments under oxidative conditions in the presence of POPC liposomes, to check whether the contribution of H^•^ atoms could lead to the formation of thiyl radicals in this case as well. In all the reactions, we could observe the promotion of *cis*/*trans* isomerization of the oleic moiety between 2.1–7.9% (Figures S9–S11), confirming that the contribution of the oxidant species leads to the regeneration of substrate **1**. 

## 3. Materials and Methods 

### 3.1. Materials

Unless otherwise noted, all commercially available compounds were used without further purification. Solvents were purchased at HPLC grade and used without further purification. Water was purified with a Millipore system. 1-Palmitoyl-2-oleoyl-sn-glycero-3-phosphocholine (POPC) was purchased from Larodan Inc. (Solna, Sweden); chloroform, methanol, diethyl ether, and n-hexane (HPLC grade) were purchased from Baker (Dover, NJ, USA). 8-TG was purchased from Santa Cruz Biotechnology Inc. (Dallas, TX, USA). Guanosine, D_2_O, and DMSO-d_6_ were purchased from Sigma Aldrich (St. Louis, MO, USA).

### 3.2. UV-Vis Absorption Spectra

The UV absorptions for the determination of the pKa were recorded with a UV-vis spectrometer Agilent Carey 100 (Santa Clara, CA, USA). Using 12 different 0.1 mM solutions of 8-TG, each at a different pH (using NaOH/phosphoric acid buffers), we recorded the absorption values and we plotted the absorption versus pH of the solution. The resulting data were fit into sigmoid curves, and the inflection points were calculated. Figure 1 was prepared with Graphpad Prism 8.0 (San Diego, CA, USA).

All other UV spectra were recorded at room temperature with a Cary300Bio spectrophotometer. 

### 3.3. HPLC-MS Analysis

HPLC-MS analyses were performed with an Agilent 1260 Infinity II HPLC system equipped with a Fortis 5 µm UniverSil C18 column (250 × 4.6 mm) using, as eluent, 0.01% formic acid in water with 0–55% gradient of acetonitrile, coupled with an InfinityLab single quadrupole liquid chromatography/mass selective detector (LC/MSD) and with a diode array detector set at 254 nm. Quantitative studies of the performed reactions were done by multiple point calibration curves (6 points) in HPLC-MS equipped with a Fortis 5 µm UniverSil C18 column (250 × 4.6 mm) using, as eluent, 0.01% formic acid in water with 0–55% gradient of acetonitrile (injection volume 5 µL, flow rate 0.7 mL/min, detection at 254 nm). Elution method: (A = H_2_O + 0.01% formic acid, B = ACN) A:B = 95:5 1 min, 45:55 at 11 min and held for 5 min, 45:55 at 15 min, back at 95:5 at 18 min, and held for 5 min. R^2^ = 0.99998 8-TG, 1.0 Guo. Retention times: 8-TG 13.02 min, Guo 9.8 min.

### 3.4. Steady-State Irradiation

Solutions (2.5 mL) of 8-TG (1.8 × 10^-4^ M) in ACN or H_2_O in a quartz cell (l = 1 cm) were irradiated using a 200 W high-pressure mercury lamp equipped with a pyrex (λ > 300 nm) or an interference filter (λ = 313 nm). For irradiation in the absence of oxygen, solutions were deaerated by bubbling with argon; otherwise, the solutions were irradiated in an open-to-air cell (air-equilibrated). In dye-sensitized photoreactions, solutions containing methylene blue or rose bengal (c = 0.05 mM) and 8-TG (1.8 × 10^−4^ M) were irradiated in air-equilibrated ACN or H_2_O solutions at >400 nm. The photoreactions were monitored by measuring the absorbance spectrum. The concentrations of substrate and photoproducts were determined by HPLC analysis. The photoproduct guanosine was identified by a comparison of the HPLC peak retention time, UV–VIS absorption, and MS spectra with a standard reference. 

### 3.5. Laser Flash Photolysis (LFP) Experiments

The set up for the nanosecond laser flash photolysis (LFP) experiments and its data acquisition system have previously been described in detail [41]. LFP experiments employed a pulsed Nd:YAG laser (λ = 266 nm, 3 mJ, 3–9 ns) for excitation. Transient decays were recorded at individual wavelengths by the step-scan method with a step distance of 10 nm in the range of 320–800 nm as the mean of 10 xenon-lamp pulses. 

### 3.6. Experiments in Gammacell

Irradiations were performed at room temperature (22 ± 2 °C) using a ^60^Co-Gammacell at different dose rates. The exact absorbed radiation dose was determined with the Fricke chemical dosimeter, by taking *G*(Fe^3+^) = 1.61 µmol·J^−1^ [43].

In the radiation experiments, 4 mL vials equipped with a rubber septum were used. Two milliliters of freshly prepared stock solutions (0.09 or 0.3 mM) of 8-TG were added together with specific amounts of additives, depending of the experiments (see above). Afterwards, the solution was degassed with either N_2_ or N_2_O for 10 min via a cannula, sealed with parafilm, and irradiated in a Gammacell. After the irradiation time, the reaction mixtures were injected in HPLC-MS without further workup.

For kinetic experiments, different reactions were prepared from a single mother solution, and then each one was stopped at a different dose. Figures were prepared with the software Graphpad Prism 8.0 and OriginLab.

For the experiments in the presence of POPC liposomes, the workup of the reactions was done as follows: 2:1 chloroform/methanol (5 × 4 mL) was added to the reaction mixture according to the Folch method [53]. The organic layer was collected and dried over anhydrous Na_2_SO_4_ then evaporated under vacuum to dryness. One milliliter of 0.5 M solution of KOH in methanol was added to each sample for transesterification of the fatty acid containing esters, which was performed by stirring for 30 min at room temperature. The methanolic reaction mixture was quenched with brine (1 mL), then the extraction was repeated with n-hexane (3 × 2 mL), and the organic phases were collected, dried over anhydrous Na_2_SO_4_, and evaporated to dryness. The fatty acid methyl ester (FAME) residue of each sample was dissolved in 50 μL of n-hexane, and 1 μL was injected for GC analysis. 

### 3.7. Preparation of 1-Palmitoyl-2-oleoyl-sn-glycero-3-phosphocholine (POPC) Liposomes

Large unilamellar vesicles by extrusion technique (LUVETs) were prepared using the hydration-extrusion technique [54]. Briefly, POPC (76 mg; 0.1 mmol) was dissolved in CHCl_3_/MeOH 2:1 until a clear lipid solution was obtained. The organic solvent was removed using a rotary evaporator to yield a homogeneous lipid film on the sides of a round-bottom flask. The lipid film was thoroughly dried to remove residual organic solvent by placing the vial or flask on a vacuum pump for 1 h. The dried lipid film was left to hydrate for 30 min in PBS and then vortexed for 10 min until a milky monophasic solution containing multilamellar vesicles (MLVs) was obtained. Once a stable suspension was formed, MLVs were downsized to LUVETs using a LiposoFast hand extrusion device (Avestin Inc., Ottawa, ON, Canada) equipped with 100 nm pore size polycarbonate filters through which the lipid suspension was passed 19 times, controlling the temperature with a heat block when required. The resulting suspension was used in the irradiation experiments. 

### 3.8. GC Analysis of Fatty Acid Methyl Esters

Fatty acid methyl esters (FAMEs) were analyzed by GC (Agilent 6850, Milan, Italy), using the split mode (50:1), equipped with a 60 m × 0.25 mm × 0.25 µm (50% cyanopropyl)-methylpolysiloxane column (DB23, Agilent, USA), and a flame ionization detector with the following oven program: temperature started at 165 °C, held for 3 min, followed by an increase of 1 °C/min up to 195 °C, held for 40 min, followed by a second increase of 10 °C/min up to 240 °C and held for 10 min. A constant pressure mode (29 psi) was chosen with helium as the carrier gas. FAMEs were identified by comparison with authentic samples, and chromatograms were examined as described previously.

### 3.9. NMR Analysis of 8-TG

NMR spectra were recorded at ambient temperature on a Varian 500 MHz spectrometer. D_2_O and DMSO-d_6_, purchased from Sigma Aldrich (St. Louis, MO, USA), were used as solvents. 

## 4. Conclusions

In conclusion, we reported a detailed study on the reactivity of biologically relevant 8-thioguanosine under oxidative and reductive conditions by using photooxidation, laser flash photolysis, and γ-radiolysis. 

Direct excitation (λ > 310 nm) of 8-thioguanosine in aerated aqueous solution gave guanosine instead of oxidized sulfur derivatives (purine sulfonic and sulfinic acids). It appears reasonable to postulate that 8-TG transformation under direct irradiation occurs with intermediacy of ^1^O_2_. The properties of the lowest energy excited state of 8-TG (T_1_) have been studied by laser flash photolysis, and it was subsequently established that this excited T_1_ state could act as a sensitizer, while in its ground state it could act as an acceptor of ^1^O_2_. Guanosine, formally a reduction product of 8-TG, is a rather unexpected photoproduct obtained from the photooxidation reaction of thiocarbonyl compounds, and singlet oxygen is a key intermediate in the desulfurization of the compound studied. Subsequent investigations on the γ-radiolysis of 8-TG highlighted a complex mechanistic scenario, which was studied by evaluating the effects of the predominant presence of each reactive species derived by the radiolysis of water. The presence of oxidant species such as HO^•^ radicals, Br_2_^•−^, and N_3_^•^ resulted, supposedly, in the regeneration of substrate **1**. Conversely, control experiments in the presence of POPC liposomes and reductive conditions displayed the possible involvement of H^•^ atoms, H_2_O_2_, and/or e_aq_^−^/H^+^ in the desulfurization of 8-TG by the generation of thiyl radicals and the simultaneous formation of the desulfurized product **2**.

## Supplementary Materials

The following are available online at https://www.mdpi.com/1420-3049/24/17/3143/s1, Figure S1: ^1^H NMR spectrum of 8-TG; Figure S2: Zoomed ^1^H NMR spectrum of 8-TG; Figure S3: ^1^H NMR spectrum of 8-TG after adding D_2_O; Figure S4: Reaction of Br_2_^•−^ with 8-TG; Figure S5: Reaction of N_3_^•^ with 8-TG; Figure S6: Reaction of 8-TG in the presence of H_2_O_2_ outside Gammacell; Figure S7: Reaction of HO^•^, H^•^, and e_aq_^−^ with 8-TG in the presence of POPC liposomes; Figure S8: Reaction of e_aq_^−^ with 8-TG in the presence of POPC liposomes; Figure S9: Reaction of HO^•^ with 8-TG in the presence of POPC liposomes; Figure S10: Reaction of Br_2_^•−^ with 8-TG in the presence of POPC liposomes; Figure S11: Reaction of N_3_^•^ with 8-TG in the presence of POPC liposomes; Figure S12: Reaction of H_2_O_2_ with 8-TG in the presence of POPC liposomes.

## Abbreviations

8-TG	8-thioguanosine
6-TG	6-thioguanosine
ROS	reactive oxygen species
Guo/G	guanosine
FAME	fatty acid methyl ester
HPLC-MS	high performance liquid chromatography mass spectrometry
GC	gas chromatography
POPC	1-palmitoyl-2-oleoyl-sn-glycero-3-phosphocholine
MB	Methylene Blue
RB	Rose Bengal
8-oxo-G	8-oxo-guanosine
