# Supplementary material for: Converging Fate of the Oxidation and Reduction of 8-Thioguanosine
Source: Molecules. 2019 Aug 29;24(17):3143. doi: 10.3390/molecules24173143 (PMC6749358; doi:10.3390/molecules24173143)
Supplement: Supplementary file 1 [file molecules-24-03143-s001.pdf]

Converging fate of the oxidation and reduction of 8-thioguanosine

Katarzyna Taras- Goslinska [1†], Fabrizio Vetica [2,3†], Sebastián Barata-Vallejo[3,4], Virginia Triantakostanti [3], Bronisław Marciniak [1,5], and Chryssostomos Chatgilialoglu [2,3,5]*

Supporting Information

1. NMR analysis 2

2. Reaction of $Br_2^{\bullet-}$ with 8-TG 4

3. Reaction of $N_3^{\bullet}$ with 8-TG 5

4. Reaction of 8-TG with H_2O_2 5

5. Reactions in the presence of POPC liposomes 6

1. NMR analysis

8-mercaptoguanosine (8-TG, 1)

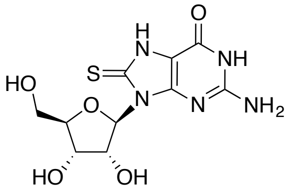

1

^{1}**H NMR** (500 MHz, DMSO-d_6) δ = δ 12.63 (s, 1H), 11.13 (s, 1H), 6.54 (s, 2H), 6.25 (d, J = 5.5 Hz, 1H), 5.24 (d, J = 5.9 Hz, 1H), 4.95 (q, J = 5.1 Hz, 1H), 4.92 – 4.80 (m, 1H), 4.77 (t, J = 5.4 Hz, 1H), 4.21 (s, 1H), 3.79 (q, J = 4.6 Hz, 1H), 3.65 (dt, J = 11.1, 4.3 Hz, 1H), 3.49 (dt, J = 11.8, 5.9 Hz, 1H). ppm. ^{1}**H NMR** (500 MHz, DMSO-d_6 + D_2O) δ = 6.25 (d, J = 5.9 Hz, 1H), 4.89 (t, J = 5.8 Hz, 1H), 4.29 – 4.23 (m, 1H), 3.88 (q, J = 4.2 Hz, 1H), 3.67 (dd, J = 12.2, 3.7 Hz, 1H), 3.56 (dd, J = 12.2, 5.1 Hz, 1H) ppm.

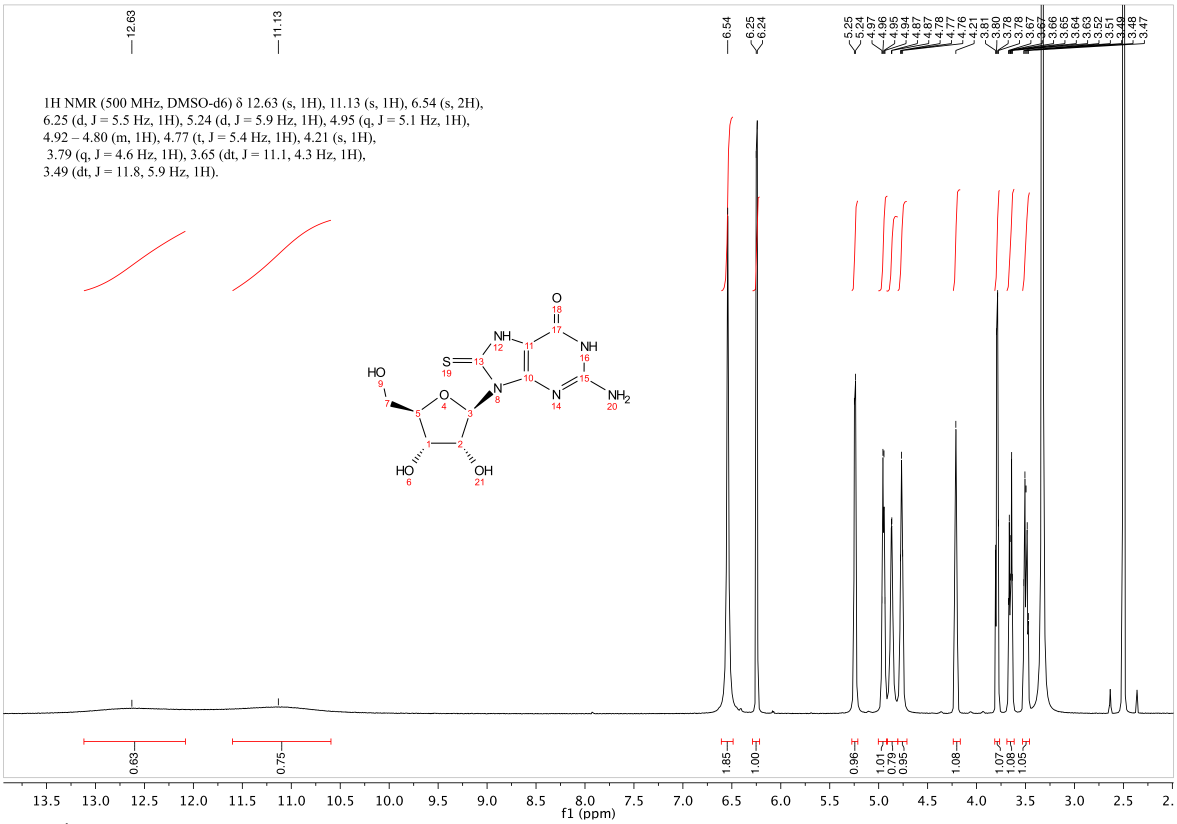

Figure S1 ^{1}H NMR spectrum of 8-TG

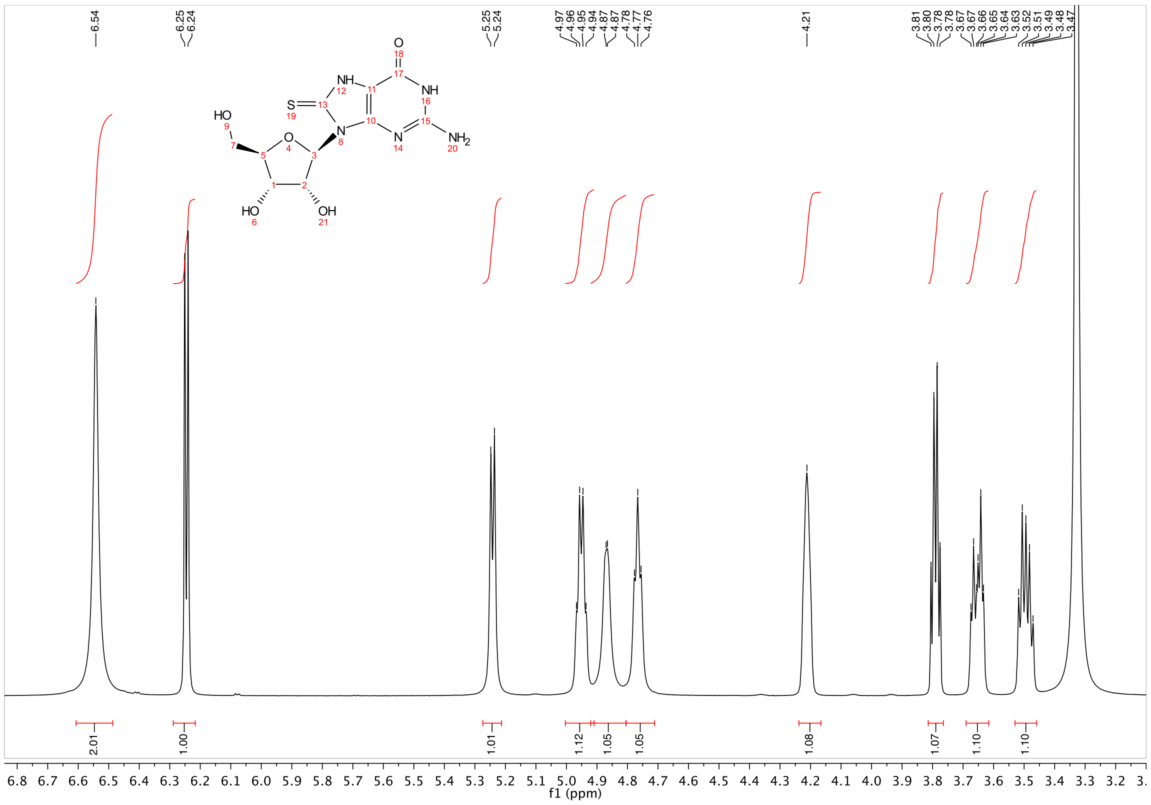

Figure S2 Zoomed ^{1}H NMR spectrum of 8-TG

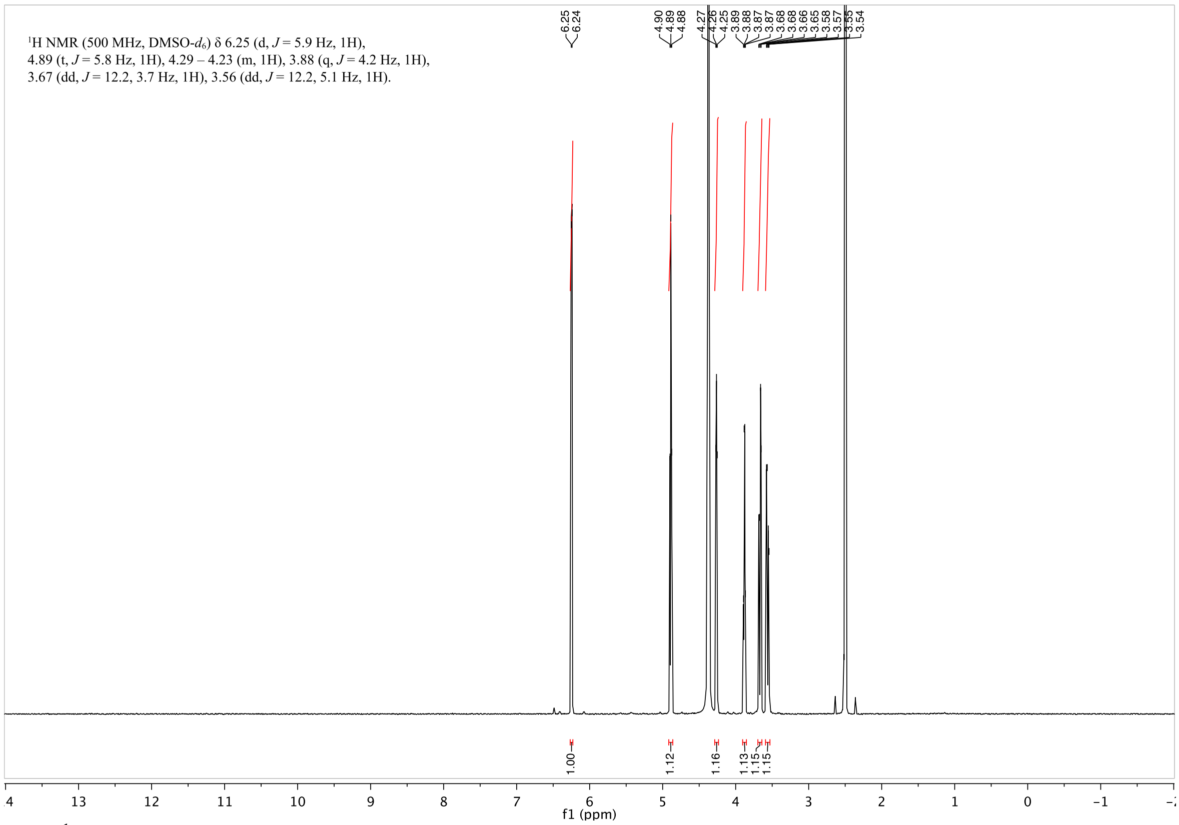

Figure S3 ^{1}H NMR spectrum of 8-TG after adding D_2O

2. Reaction of $Br_2^{\bullet-}$ with 8-TG

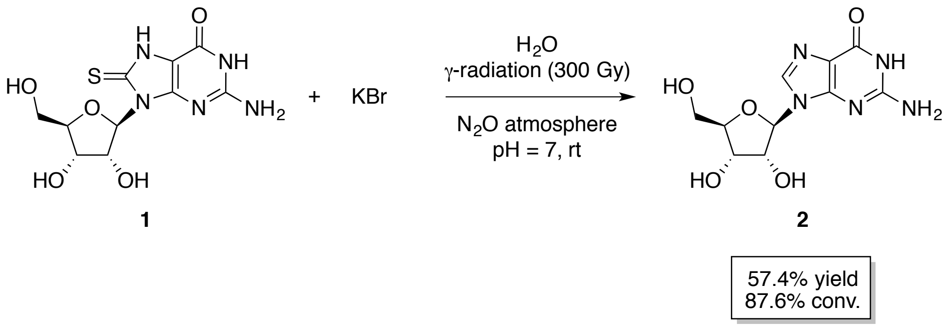

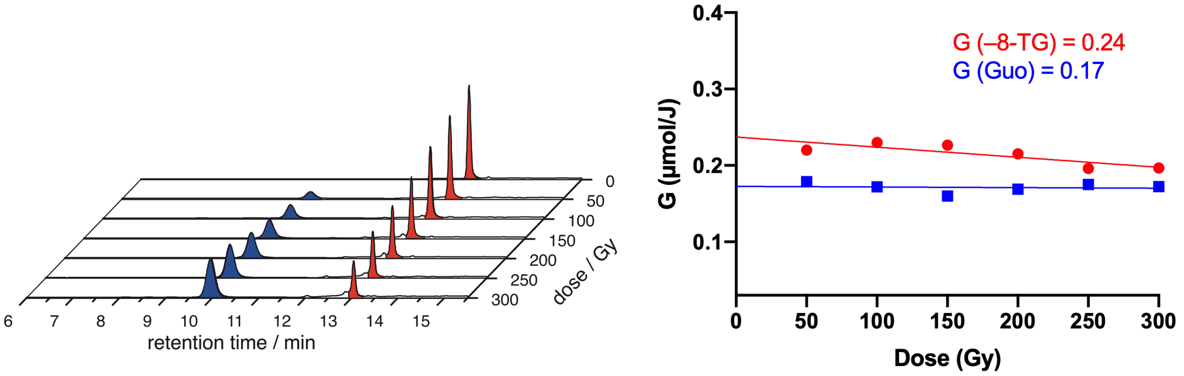

Figure S4 Reaction of $Br_2^{\bullet-}$ with 8-TG. γ-radiolysis of **1** (0.09 mM) in N_2O-purged water at natural pH, containing KBr (0.1 M) at a dose rate of 1.85 Gy min^{-1}. A) HPLC runs of the reactions. The HPLC peaks of **1** are hihlighted in red, while the peaks of guanosine (**2**) are highlighted in blue. B) The chemical irradiation yields *G* (–**1**) (●) and *G* (**2**) (■) as function of the irradiation dose. The line extrapolation to a zero dose leads to the *G* values reported on the graph.

3. Reaction of $N_3^{\bullet}$ with 8-TG

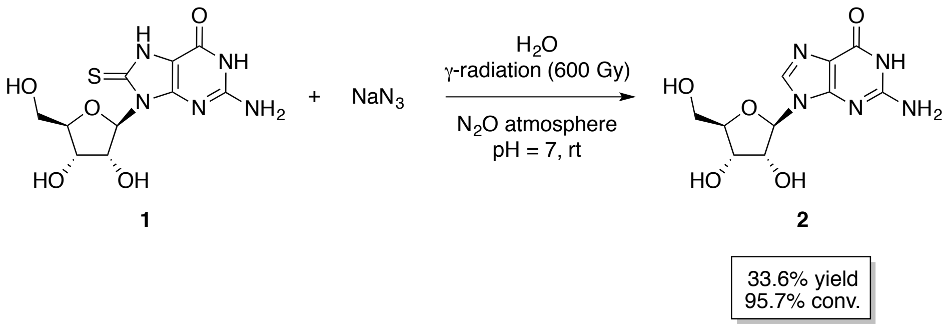

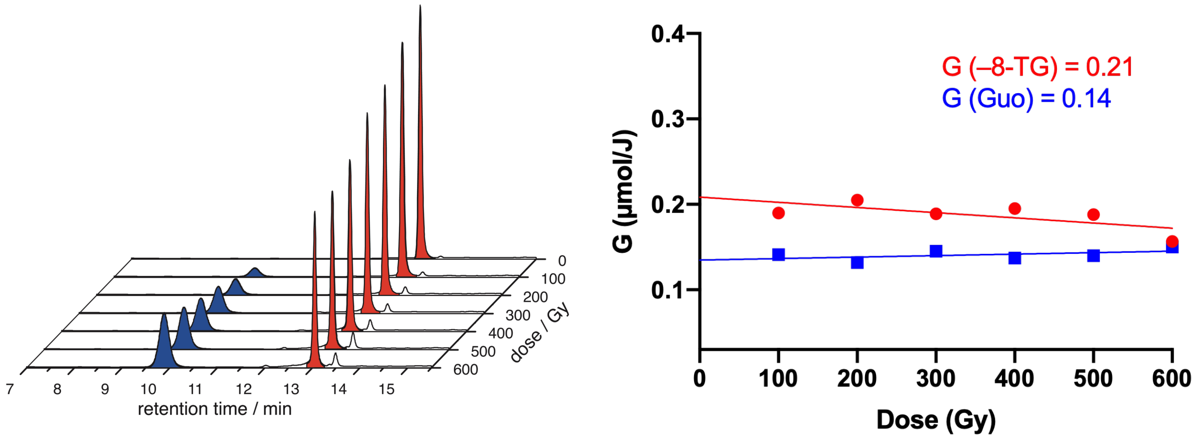

Figure S5 Reaction of $N_3^{\bullet}$ with 8-TG. γ-radiolysis of **1** (0.27 mM) in N_2O-purged water at natural pH, containing NaN_3 (0.1 M) at a dose rate of 1.85 Gy min^{-1}. A) HPLC runs of the reactions. The HPLC peaks of **1** are hihlighted in red, while the peaks of guanosine (**2**) are highlighted in blue. B) The chemical irradiation yields *G* (–**1**) (●) and *G* (**2**) (■) as function of the irradiation dose. The line extrapolation to a zero dose leads to the *G* values reported on the graph.

4. Reaction of 8-TG with H_2O_2

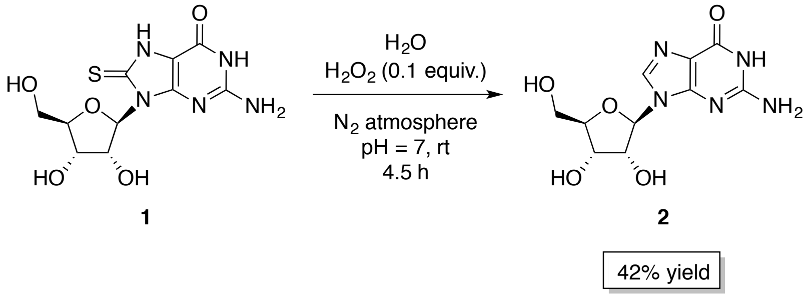

Figure S6 Reaction of 8-TG in the presence of H_2O_2 outside Gammacell.

8-TG (0.3 mM) and H_2O_2 (30% water solution, 0.1 equiv.) were dissolved in 2 mL water. The solution was saturated with N_2 for 7 min and left without stirring for 4.5h (corresponding to an irradiation of 500 Gy if the reaction was carried out in Gammacell). After the irradiation time, the solution was analysed *via* HPLC.

5. Reactions in the presence of POPC liposomes

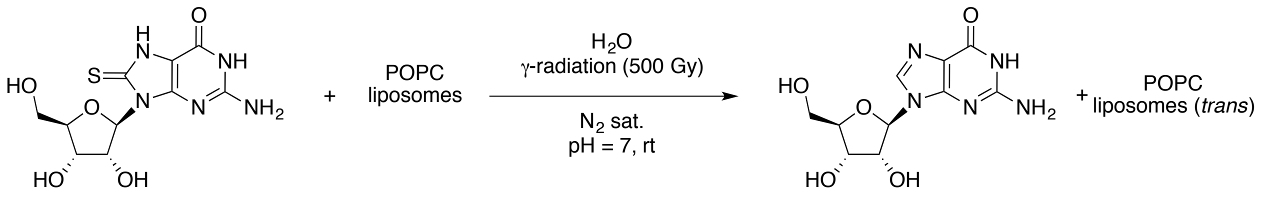

1 0.3 mM — 2 mM — **2**

4.5% isomerization

Figure S7 Reaction of HO•, H•, and e_{aq}^{-} with 8-TG in the presence of POPC liposomes. γ-radiolysis of **1** (0.3 mM) in N_2-purged water at natural pH, containing POPC liposomes (2 mM), at a dose rate of 1.85 Gy min^{-1}. The level of isomerization was determined *via* GC analysis.

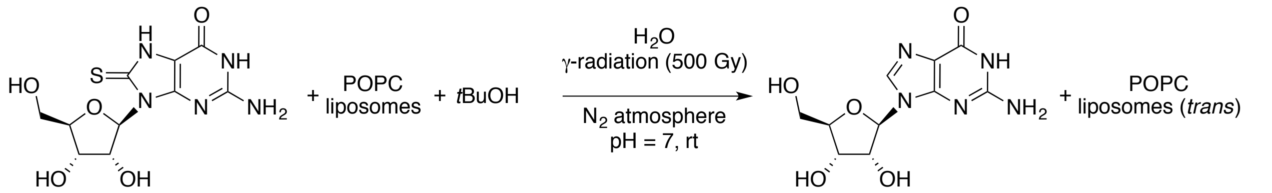

1 0.3 mM — 2 mM — 0.250 M — **2**

13.7% isomerization

Figure S8 Reaction of e_{aq}^{-} with 8-TG in the presence of POPC liposomes. γ-radiolysis of **1** (0.3 mM) in N_2-purged water at natural pH, containing POPC liposomes (2 mM) and *t*BuOH (0.25 M), at a dose rate of 1.85 Gy min^{-1}. The level of isomerization was determined *via* GC analysis

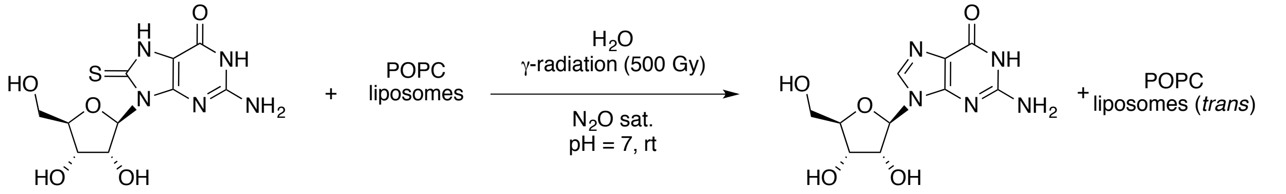

1 0.3 mM — 2 mM — **2**

7.9% isomerization

Figure S9 Reaction of HO• with 8-TG in the presence of POPC liposomes. γ-radiolysis of **1** (0.3 mM) in N_2O-purged water at natural pH, containing POPC liposomes (2 mM), at a dose rate of 1.85 Gy min^{-1}. The level of isomerization was determined *via* GC analysis.

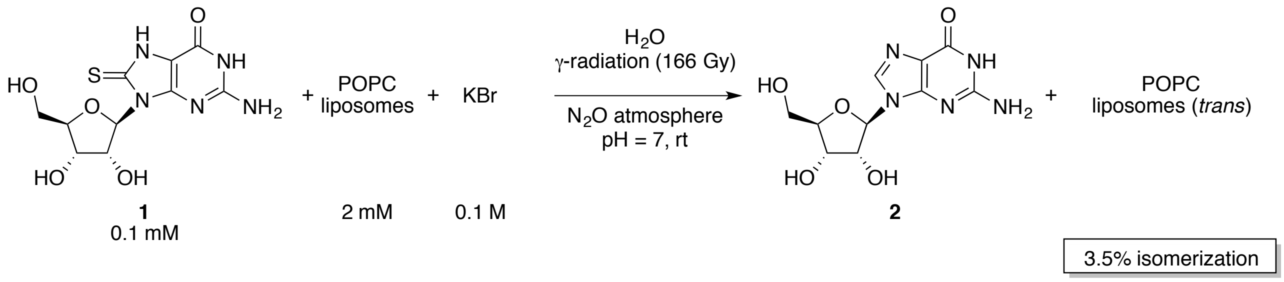

Figure S10 Reaction of $Br_2^{\bullet-}$ with 8-TG in the presence of POPC liposomes. γ-radiolysis of **1** (0.1 mM) in N_2O-purged water at natural pH, containing POPC liposomes (2 mM) and KBr (0.1 M), at a dose rate of 1.85 Gy min^{-1}. The level of isomerization was determined *via* GC analysis.

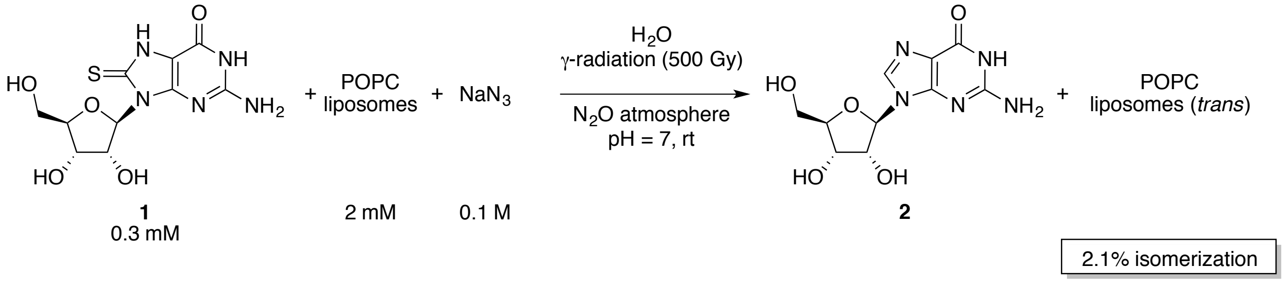

Figure S11 Reaction of $N_3^{\bullet}$ with 8-TG in the presence of POPC liposomes. γ-radiolysis of **1** (0.3 mM) in N_2O-purged water at natural pH, containing POPC liposomes (2 mM) and NaN_3 (0.1 M), at a dose rate of 1.85 Gy min^{-1}. The level of isomerization was determined *via* GC analysis.

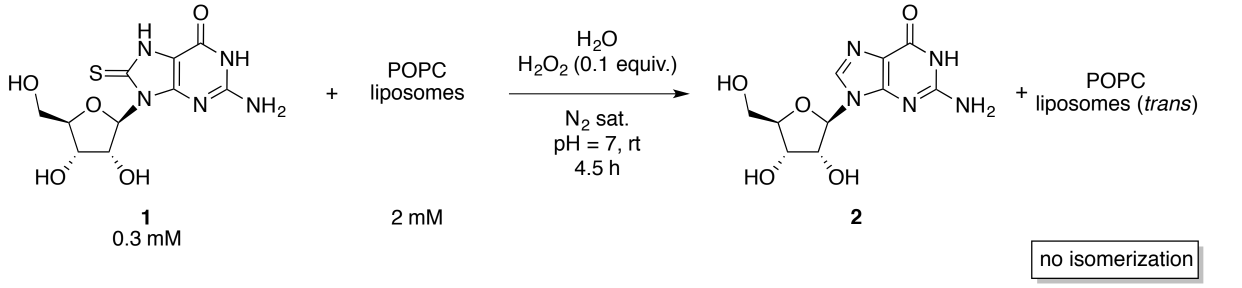

Figure S12 Reaction of H_2O_2 with 8-TG in the presence of POPC liposomes. Reaction of **1** (0.3 mM) and H_2O_2 (0.1 equiv.) in N_2-purged water at natural pH, containing POPC liposomes (2 mM), at a dose rate of 1.85 Gy min^{-1}. The level of isomerization was determined *via* GC analysis.
